# Optical performance of minimally invasive brain implants: a simulation and phantom study

**DOI:** 10.1364/BOE.604502

**Published:** 2026-08-12

**Authors:** Vytautas Gradauskas, Jemma Neil, Kevin J. Mitchell, Jack Radford, Daniele Faccio

**Affiliations:** School of Physics and Astronomy, University of Glasgow, Glasgow, UK

## Abstract

Despite significant progress in non-invasive techniques to sense brain activity through highly scattering biological tissue, these modalities are inherently limited by the physical process of diffusion. We explore minimally invasive transdermal and transcranial optical implants that directly reduce light diffusion by bypassing highly scattering superficial tissues, and quantitatively evaluate their impact on detected photon energy and sensitivity profiles to provide engineering insights for the optimisation of optical interface design and placement. Simulations using an anatomical head model show increased energy detection by 1-4 orders of magnitude, with improved cerebral cortex sensitivity by up to 3 orders of magnitude for longer SDS. Optical implants for SDS of a few millimetres also enable the probing of cerebral cortex with high ∼1 mm localisation. Experiments with an anatomically correct (based on an MRI scan) but simplified resin-based phantom validate the numerical modelling and show good agreement with the simulated detected energies. Further development of these optical implants could be an option for longitudinal brain sensing and imaging measurements.

## 1. Introduction

Functional near-infrared spectroscopy (fNIRS), as well as diffuse optical tomography are techniques used in neuroscience and psychology, biomedical imaging and computer-brain interface research [1–3]. These techniques rely on measuring photons that have propagated through multiple layers of highly scattering tissue. The placement of a spatially separated (typically 5-50 mm [4,5]) source and detector on the head results in a characteristic banana-shaped sensitivity profile of light that is collected for detection scatters through several anatomical layers (e.g. scalp, skull, cerebrospinal fluid, gray and white matter). The larger the source-detector separation (SDS), the deeper the sensitivity profile penetrates into the head, and in general, the better the overlap with the gray matter (GM) where the relevant brain activity occurs. On the other hand, the amount of light collected at the detector decreases exponentially as the source-detector separation increases – consequently, the measured signal degrades very quickly with SDS.

Unfortunately, light loss cannot be compensated for by simply increasing laser power, as whilst the decrease is exponential with distance, increasing laser power only provides a linear increase in signal. In addition, there are laser power safety limits in human tissue that set an upper limit. On the other hand, diffuse light can be guided through low-scattering channels (core) that are surrounded by high-scattering regions (cladding) [6]. Within the human head, the cerebrospinal fluid (CSF) layer may act as a weakly scattering core, enclosed by highly scattering gray/white matter and extracerebral layers (skull and scalp). This structural configuration enables light to propagate over relatively long distances, e.g., diametrically across an adult human head [7], prior to detection.

Ultra-high-density diffuse optical tomography (UHD-DOT) and time-domain (TD) fNIRS approaches aim to address the same fundamental challenge: improving localised sensitivity to GM while reducing the influence of superficial tissues. UHD-DOT relies on dense spatial sampling to better characterize photon migration across multiple source-detector separations [4] and employs short-channel regression to suppress superficial physiological signals during post-processing. In contrast, TD-fNIRS exploits the time-of-flight distribution of short laser pulses, particularly through early- and late-gated measurements, to improve depth selectivity and enhance the separation of cerebral and extracerebral contributions [8,9]. Furthermore, null- and near-null source-detector separation geometries have been shown to improve inclusion contrast and localisation [10,11]. Despite these advances, all non-invasive optical approaches remain fundamentally constrained by the exponential attenuation of light through the scalp and skull. As a result, only a limited fraction of light interrogates the brain, of which then only a fraction is actually collected. Increasing channel density provides additional spatial sampling but does not increase the amount of light returning from the cortex in each individual measurement, while time-gating increases the relative contribution of late-arriving, brain-sensitive photons without increasing the total number of detected cortical photons. Neither of these options provide complete elimination of the overwhelming superficial signal contamination. In contrast, minimally invasive optical interfaces bypass these extracerebral layers, fundamentally overcoming this attenuation and contamination limit rather than mitigating its effects.

Previous studies have demonstrated the feasibility of invasive transcranial fNIRS experiments on primates with removed sections of scalp and bone [12,13]. More recently, Rein et al. introduced an optical anchor bolt (OAB) that could enable simultaneous intracranial electroencephalography and fNIRS recordings [14]. Their study combined Monte Carlo (MC) simulations based on a slab model experimental validation, demonstrating increased detected signal and sensitivity to the cerebral cortex.

Similar improvements in optical access may also be achievable without physical implants. For example, tissue transparency can be induced by applying tartrazine dye or alcohols that alter the optical properties of skin or bone [15–17], allowing injected light to penetrate more effectively. In principle, such agents could be locally applied or injected into the scalp to enhance transparency, although current effects are temporary and typically last only 10–20 minutes before the tissue must be washed.

In this work, we systematically investigate multiple configurations involving small diameter optical implants that extend either transdermally (through the scalp only) or transcranially (through both scalp and skull), compared and quantified their effects on light detection efficiency and sensitivity. We conducted numerical simulations on an open source MRI-derived head model with accurate anatomical structure. The implants enable the probing of GM with SDS of only a few millimetres, resulting in up to 5 orders of magnitude increased sensitivity. The overall increased sensitivity to GM can be detected with up to 4 orders of magnitude higher efficiency. We also validated the numerical model experimentally using a three-layer 3D white resin-printed head phantom consisting of shell (scalp and skull combined), cerebrospinal fluid (CSF), brain layers derived from an MRI scan also used in simulations.

## 2. Methods

**Fig. 1.**
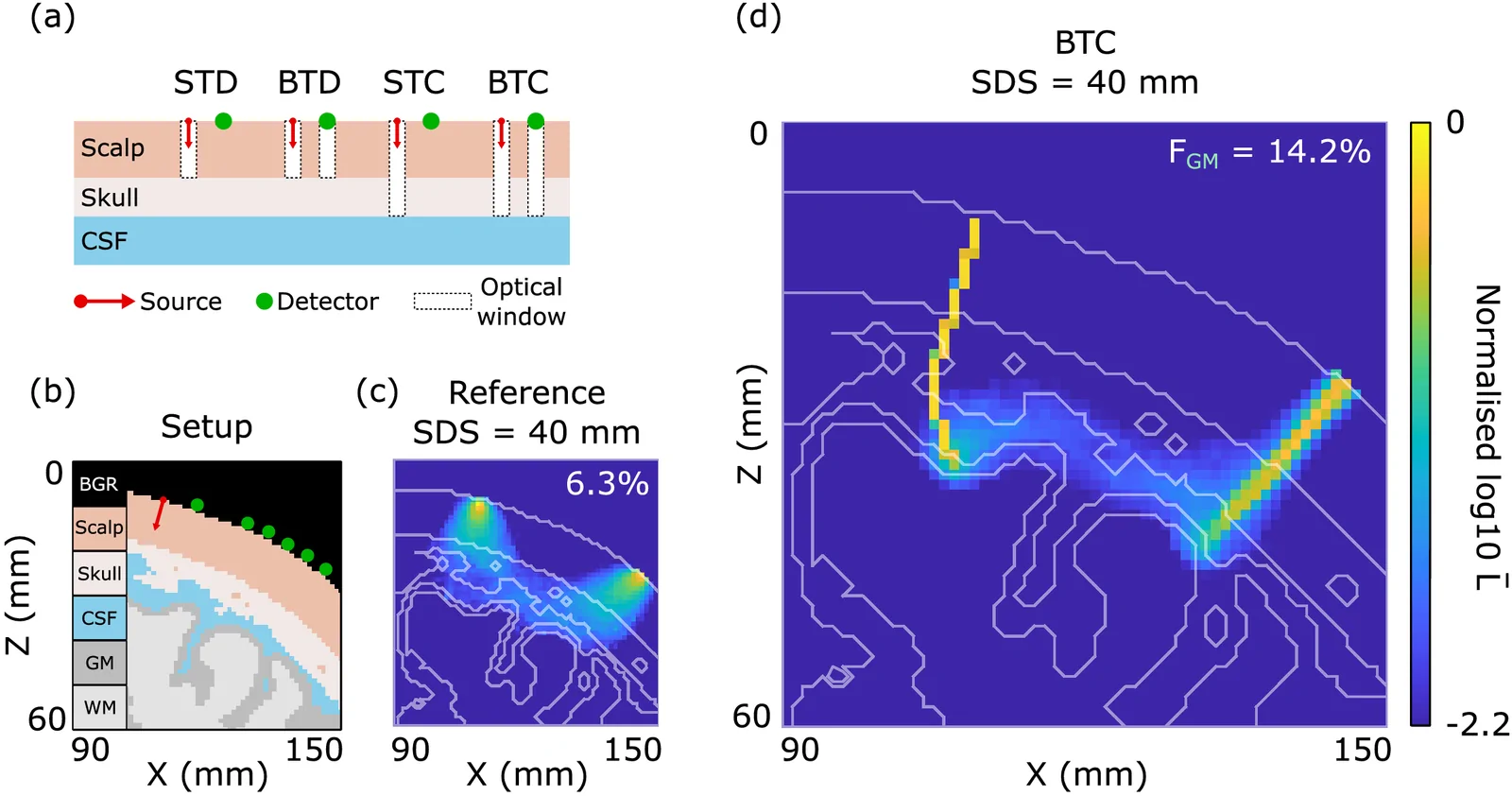
A 2D graphical sketch of different transdermal and transcranial configurations (a). The abbreviations describe optical implants (white regions with dotted outline) for when the source alone is transdermal (STD), both source and detector are transdermal (BTD), the source is transcranial (STC) and both source and detector are transcranial (BTC) cases. Red arrows indicate the source position and direction. The green circles indicate detector positions. (b) The simulation setup showing the Colin27 head atlas cross section with different tissue types. The green circles mark the positions of 6 spherical detectors placed at SDS = 8, 20, 25, 30, 35, 40 mm. (c) Normalised logarithmic spatial sensitivity profile for SDS = 40 mm for the standard case and in (d), for the BTC case (both source and detector with transcranial implants). The percent values in (c and d) indicate fractional GM sensitivity $\left(F_{GM}\right)$ as defined by Eq. (3). The colorbar is common for (c-d) spatial sensitivity maps and shows 2.2 orders of magnitude from the maximum value to enhance the sensitivity profile shapes.

Our numerical model was built using the Monte Carlo ray-tracing light transport method – for this we used Monte Carlo eXtreme (MCX) [18]. For the first simulations presented we used the open source adult human head volume atlas Colin27 [19,20], which consists of six layers: scalp, skull, CSF, gray matter, white matter and air pockets. The Colin27 model was modified to simulate transdermal and transcranial implants – this was achieved by modelling simplified glass (n=1.5) implants as cylindrical optical windows with diameter $d_{\text{implant}}≃2\;\text{mm}$ (approximated due to head atlas voxelization with isotropic 1 mm voxels). The optical implants were positioned normal to the surface of the head atlas. Transdermal optical implants penetrate only through the scalp depth, whilst the transcranial optical implants penetrate through the scalp and skull layers. A sketch illustrating transdermal and transcranial optical implant geometry is shown in Fig. 1(a).

A total of five regimes were investigated: firstly, an unmodified Colin27 model, henceforth referred to as the standard reference case, followed by source-transdermal (STD) and source-transcranial (STC) regimes, i.e. only the source had an optical implant. We also investigated the case for which the source and the detector both had optical implants, i.e. both-transdermal (BTD) and both-transcranial (BTC) regimes.

There is significant uncertainty regarding the true in vivo optical properties for living human tissue [21,22]. Therefore, we selected and compiled values for visible (VIS) and near-infrared (NIR) wavelengths based on well-established and widely cited Refs. [22–24]. More details on the full spectra of the adopted optical coefficients can be found in the Supplement 1. Here we used optical parameters taken at the typical fNIRS wavelength λ=850nm : the corresponding parameter values used for the simulations are displayed in Table 1.

**Table 1.** Optical coefficients for *λ* = 850 nm used in this study

Tissue type	*μ_a_* (mm^−1^)	$\mu_{s}^{\prime }\;({\text{mm}}^{-1})$	*n*
Scalp	0.046	2.19	1.37
Skull	0.014	1.26	1.37
CSF	0.0043	0.01	1.37
GM	0.023	1.16	1.37
WM	0.024	3.47	1.37
Air	0	0	1

**Fig. 2.**
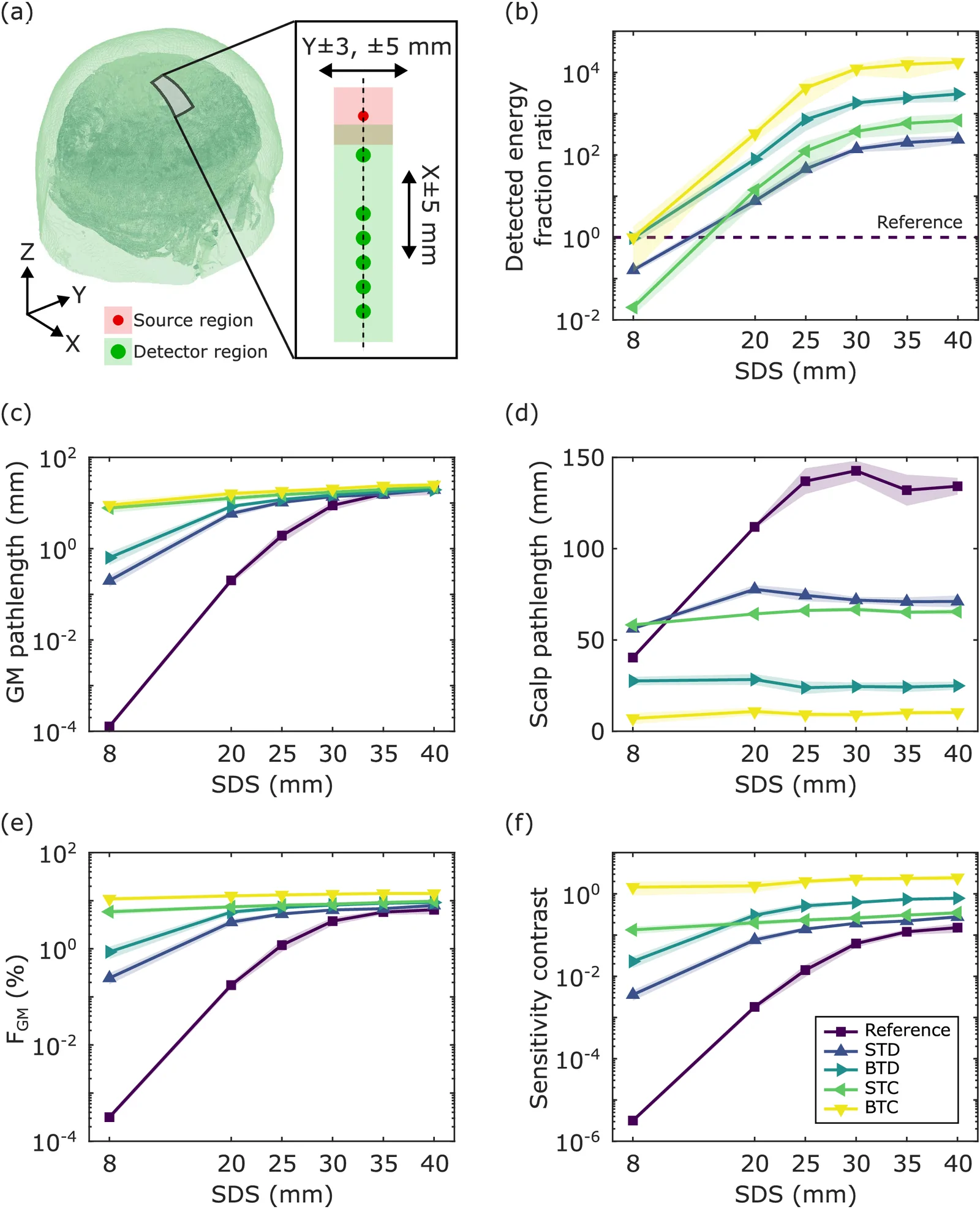
(a) 3D image of Colin27 model with a gray shaded area indicating a region where the linear montage was shifted in X- and Y-axes to consider anatomical uncertainty. The zoomed-in inset shows the linear montage (along the dashed line) consisting of source (red sphere) and 6 detectors (green spheres) spaced with SDS = 8, 20, 25, 30, 35, 40 mm. The linear montage was shifted X=±5mm and Y=±3,±5mm ,which are indicated by the shaded areas. (b) Detected energy fraction ratio as defined by Eq. (1). The dashed line is the reference case, which equals to 1. (c,d) are gray matter and scalp pathlength, respectively. (e) Fractional gray matter sensitivity as defined by Eq. (3). (f) Sensitivity contrast as defined by Eq. (4). The shaded areas in (b-f) is 1 standard deviation from the mean calculated from shifting the linear montage. The legend is common for (b-f) plots.

A cross section of the Colin27 atlas with different tissue types and the simulation setup is shown in Fig. 1(b). For each simulation a collimated pencil-beam source was angled towards the middle of the brain and placed at location C2 in the International 10-20 EEG system. Six spherical detectors with 3 mm diameter were placed on the surface of the head for different SDS. One detector was considered short-channel with a distance of 8 mm, and the other five were linearly separated by 5 mm starting from SDS = 20 mm to SDS = 40 mm. This linear montage was shifted X=±5mm and Y=±3,±5mm , as shown in Fig. 2(a). This allowed to account for anatomy-induced variations in the detected signals and analysed metrics.

### 2.1. Metrics

The quantitative evaluation of the optical implant performance was carried out using metrics commonly employed in diffuse biomedical optics [14,25]. We define the detected energy fraction ratio as:

(1) $$E_{\text{ratio}}=\frac{E_{x}}{E_{\text{ref}}},$$

where $E_{x}$ is the detected energy fraction for a given implant configuration, and $E_{\text{ref}}$ is the corresponding value for the reference case.

In the context of MCX simulations, the detector records the sum of attenuated photon packet weights. For a normalised simulation in which *N_p_* photon packets are launched, each with initial weight of $w_{0}=1$ , the detected energy fraction is defined as:

(2) $$E=\frac{\sum w_{\text{det}}}{N_{p}w_{0}},$$

where $\sum w_{\text{det}}$ is the sum of photon weights recorded by the detector. This quantity represents the fraction of the total launched energy reaching the detector. If a physical energy scale is required, the detected energy can be obtained by multiplying this fraction by the total source energy $E_{\text{src}}$ .

We derive the fractional GM sensitivity [25] for each implant and reference case as:

(3) $$F_{GM}=\frac{{\bar{L}}_{GM}}{{\bar{L}}_{\text{total}}}\times 100\%,$$

where ${\bar{L}}_{GM}$ is the weight-averaged partial pathlength of detected photon packets within the gray matter, and ${\bar{L}}_{\text{total}}$ is the total weight-averaged pathlength across all tissue types. The $F_{GM}$ metric represents the fraction of the detected photon pathlength that is spent within gray matter, and thus quantifies the overall sensitivity of the measurement to cerebral tissue.

We use the definition from [14] to introduce sensitivity contrast as:

(4) $$\text{Sensitivity contrast}=\frac{{\bar{L}}_{GM}}{{\bar{L}}_{\text{scalp}}},$$

where ${\bar{L}}_{\text{scalp}}$ is the weight-averaged partial pathlength in the scalp. While $F_{GM}$ captures the absolute contribution of gray matter relative to all tissues, sensitivity contrast characterizes the relative balance between cerebral and superficial contributions.

Together, these metrics provide complementary information: $F_{GM}$ reflects the overall sensitivity to brain tissue, whereas sensitivity contrast indicates the extent to which this sensitivity is preferentially weighted toward gray matter over the superficial layer. As such, sensitivity contrast can be interpreted as a proxy for robustness against superficial signal contamination, although it does not constitute a formal signal-to-noise ratio, as no explicit noise model is included.

### 2.2. Experimental validation

In order to validate the numerical simulations, we also performed experiments on a simplified head phantom: the goal here is not to reproduce all of the results above but rather just to verify that numerical simulations and experiments provide similar results in a fixed setting. A 3D resin-printed adult human head phantom was created from a single 3T MRI scan, segmented into three layers - brain, CSF and shell (skull and scalp combined). We 3D-printed the shell and brain volumes using Formlabs Form 3 printer with white resin V4.1. The cured resin was characterised using a time-of-flight measurement (see Supplement 1). We measured the reduced scattering coefficient as $\mu_{s}^{\prime }≃4\;{\text{mm}}^{-1}$ and the absorption coefficient as $\mu_{a}≃0.001\;{\text{mm}}^{-1}$ . The phantom was completed by introducing a low-loss gelatin layer mimicking CSF between the shell and brain. This last step was completed by submerging the whole 3D print in gelatine and once the gelatine cured, the excess was removed, resulting in the final simplified 1:1 scale adult human head phantom (Fig. 4 (a)).

The implant measurements were taken for SDS = 8, 20, 25, 30, 35, 40 mm. The source was a λ=685nm wavelength continuous wave fibre-coupled laser (Thorlabs MCLS1-685). This was coupled to a modified single-mode fibre (Thorlabs P1-630A-FC-1) that was inserted into a hypodermic needle (Fig. 4(b)), which in turn was inserted into 1 mm-diameter, 5 mm-deep source holes drilled into the phantom. The source power was kept constant to 3.7 mW throughout the experiments. We then used a 1.5 mm core diameter, 0.5 NA multi-mode fibre (Thorlabs M107L01) for detection which was coupled to a Thorlabs S121C power sensor connected to PM400 power meter head. Standard reference measurements were obtained by placing the source fibre on the surface of the phantom before the source holes were drilled into the resin.

We numerically replicated the experimental measurements with an MCX simulation using the same segmented three-layer head model used for 3D printing. The optical coefficients for the shell and brain were set to be the measured values of $\mu_{a}=0.001\;{\text{mm}}^{-1},\mu_{s}^{\prime }=4\;{\text{mm}}^{-1}$ and refractive index n=1.505 as measured by [26]. The low-loss CSF layer was modelled with a 100 times lower scattering coefficient, keeping the other optical coefficients the same.

Figure 4(c) shows a close-up of the simulation setup for the optical implant case. A pencil beam source was placed on the surface of the model pointing towards the middle of the brain. We placed six spherical detectors with 3 mm diameter in a line with short channel SDS = 8 mm and the other five linearly separated by 5 mm starting from SDS = 20 mm to SDS = 40 mm. Although we carefully replicated the experimental optode placement in our simulations, we also introduced controlled variability to account for potential placement differences. We shifted the entire montage (source and detectors) by X=±5mm and Y=±3mmand±5mm , generating multiple simulations. For each of the 7 montage positions, $10^{9}$ photon packets were simulated on a Windows 10 machine with NVIDIA GeForce RTX 4090. All simulations required approximately 12 minutes to complete.

## 3. Results

The MCX (v2024.2) simulations were performed on a Windows 10 machine with NVIDIA GeForce RTX 4090 graphics processing unit. A number of $10^{9}$ photon packets were launched for each linear montage position (7 positions) and for each case (5 total cases – 1 reference and 4 implants cases). This resulted in approximately 10 hours and 50 minutes of total simulation time for a total number of $5\times 7\times 10^{9}=3.5\times 10^{10}$ launched photon packets.

### 3.1. Sensitivity profiles

In order to first provide an intuitive picture of the impact of the implants, we show as an example the normalised logarithmic spatial sensitivity profiles for SDS = 40 mm, Fig. 1(c-d) (see Supplement 1 for other SDS cases). In the standard reference case, Fig. 1(c), we see the characteristic "banana" shape sensitivity profile that probes the gray matter with a characteristic diffuse cloud, resulting in a fractional gray matter sensitivity, as defined by Eq. (3), $F_{GM}=6.3\%$ . However, transcranial implants at both the source and detector side, BTC, modify light propagation not only through the scalp/skull but also in the gray matter region, Fig. 1(d). Light is now more tightly confined by the guiding properties of the CSF and consequently, the fractional gray matter sensitivity increases by more than 2× to $F_{GM}=14.2\%$ . This increase in $F_{GM}$ is significant but still relatively modest when compared to the much higher 100× gains that are observed e.g. for short channel SDS=8 mm, as discussed in detail below.

### 3.2. Metrics

Figure 2(b) shows a comparison of the detected energy fraction ratios, $E_{\text{ratio}}$ . The absolute detected energy fractions for all cases are provided in the Supplement 1. The detected energy fraction ratio increases monotonically with SDS for all configurations. Perhaps counterintuitively, at small separations (SDS = 8 mm), all implant cases yield low collected energy fraction, with $E_{\text{ratio}}<1$ and deeper source implants also have lower detected energy ratio (the STC ratio $E_{\text{ratio}}=0.02$ is an order of magnitude lower than STD, $E_{\text{ratio}}=0.16$ ). As SDS increases, the BTC case shows the highest signal collection, rising to roughly 4 orders of magnitude above the reference case and saturating beyond ≥30mm . At these separations, the detected energy ratios plateau for all regimes. The transdermal cases are <10 times lower than their respective transcranial cases.

Although the reduction in tissue is approximately equal for source and detector optical implants, there is an asymmetric gain in detected energy fraction. At SDS of 40 mm, the $E_{\text{ratio}}$ with a single source-side implant increased substantially by 2 orders of magnitude for the STD case and by up to 3 orders of magnitude for the STC case. Removing tissue at the detector side (adding detector implant) improves the detected energy fraction additionally by 1 order of magnitude in each case.

Figure 2(c) shows gray matter sensitivity expressed as weight-averaged partial pathlength in the gray matter layer $\left(L_{GM}\right)$ . All curves monotonically increase with longer SDS and converge to a similar value of $L_{GM}≃20\;\text{mm}$ at SDS = 40 mm. The optical implants at SDS = 8 mm result in GM pathlength values comparable to those seen in the standard reference case at SDS = 20-30 mm.

Figure 2(d) reveals that the optical implants lower the scalp pathlength by 2-10× for SDS ≥ 20 mm. At SDS = 8 mm, the source optical implants (STD and STC) have ∼1.4× higher scalp pathlength than the reference case. Both transdermal case has ∼1.5× and both transcranial has ∼5.7× lower scalp pathlength than the reference case for SDS = 8 mm. All of the optical implant regimes have relatively uniform scalp pathlength over the simulated SDS, while the $L_{\text{scalp}}$ increases for the standard reference case. We observe a small dip in the scalp pathlength around SDS = 30 mm for the standard reference case. When changing the orientation for the source-detectors along the scalp, the shape of this curve, including the presence of the dip itself is modified (see Supplement 1), therefore hinting that this dip is linked to the specific skull (and overlaying scalp) shape and geometry along the source-detector direction.

The fractional gray matter sensitivity as defined by Eq. (3) is shown in Fig. 2(e). The reference case has the lowest sensitivity to gray matter for all SDS with the lowest value of $F_{GM}=0.0003\%$ at SDS = 8 mm. For typical source-detector separations used in fNIRS (SDS = 20-40 mm), the fractional GM sensitivity is in the range of *F_GM_* = 0.2-5.8%. These fractional GM sensitivity values are already achieved at SDS = 8 mm for all optical implant cases, with the transdermal implants ranging *F_GM_* 0.2-0.9% and the transcranial cases *F_GM_* 5.9-10.9%. For the longest SDS = 40 mm, the optical implant provide ∼2× increase in brain sensitivity compared to the standard reference case.

Figure 2(f) shows the sensitivity contrast, as defined by Eq. (4). This generally increases with SDS as the source and detector are separated further. The standard reference case exhibits low contrast at short separations (SDS = 8 mm), implying that the propagation of light from the source to the detector is predominately through the scalp layer. The contrast increases by up to 5 orders of magnitude with increasing SDS but remains the lowest compared to all implant configurations considered here. The highest gain in sensitivity contrast compared to the reference case is for the shortest channels – up to 3-4 orders of magnitude for the transdermal regimes and up to 5-6 orders of magnitude for transcranial regimes. The transdermal cases show a gradual improvement in sensitivity contrast with increasing SDS – the STD and BTD cases plateau after SDS ≥25mm and is up to an order of magnitude higher than the reference case. The BTC configuration yields the highest sensitivity contrast across all SDS, and remains close to a value of one, indicating that photon path lengths in GM become comparable to or exceed those in the scalp.

### 3.3. Localisation of gray matter sensitivity for short-channel implants

**Fig. 3.**
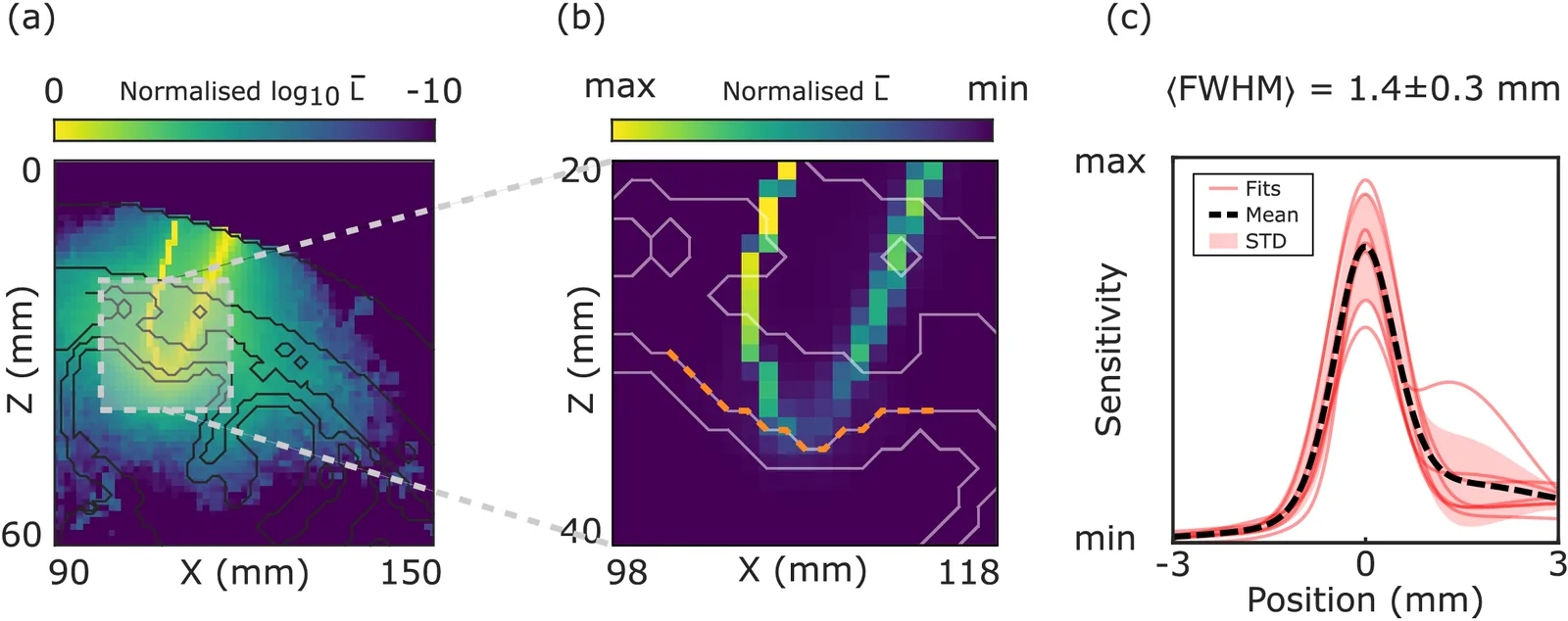
(a) Normalised logarithmic spatial sensitivity profile for SDS = 8 mm for both source and detector transcranial implant case. Colorbar shows 10 orders of magnitude from the maximum value. The dashed gray rectangle indicates a zoomed-in region shown in (b). Note that (b) has a linear colormap and without a threshold. The orange dashed line indicates the surface of the gray matter along which we sample the sensitivity. (c) Fitted and peak-aligned sensitivity profiles for each montage position (red solid lines), the average sensitivity profile (black dashed line) and standard deviation of the fitted curves (red shaded area). The mean sensitivity diameter, measured at the full width half maximum of the dashed line is ⟨FWHM⟩=1.4±0.3mm .

The highest sensitivity region for the BTC case for SDS = 8 mm in Fig. 3(a) follows a U-shape and suggests a highly localised sensitivity area on the gray matter. To investigate this, we extracted GM pathlength values along the surface of GM and then repeated this for various montage positions in order to also estimate the dependency on specific location of the implants. One example is shown in Fig. 3(b): the orange dashed line follows the gray matter boundary and the pathlength value is recorded along this line. Each extracted sensitivity profile was fitted with a double Gaussian curve. The full width at half maximum was evaluated for each curve, which we define as the diameter of the gray matter sensitivity region. The fitted and peak-aligned sensitivity profiles are shown in Fig. 3(c). The average full width at half maximum of the sensitivity-ROIs is ⟨FWHM⟩=1.4±0.3mm .

We underline that this is not the same as imaging resolution. This evaluation is based only on a single channel (averaged over 7 linear montage positions). This is therefore simply the diameter of the gray matter sensitivity region below the source implant when the separation between source and detector is small, i.e., SDS = 8 mm.

### 3.4. Experimental validation

**Fig. 4.**
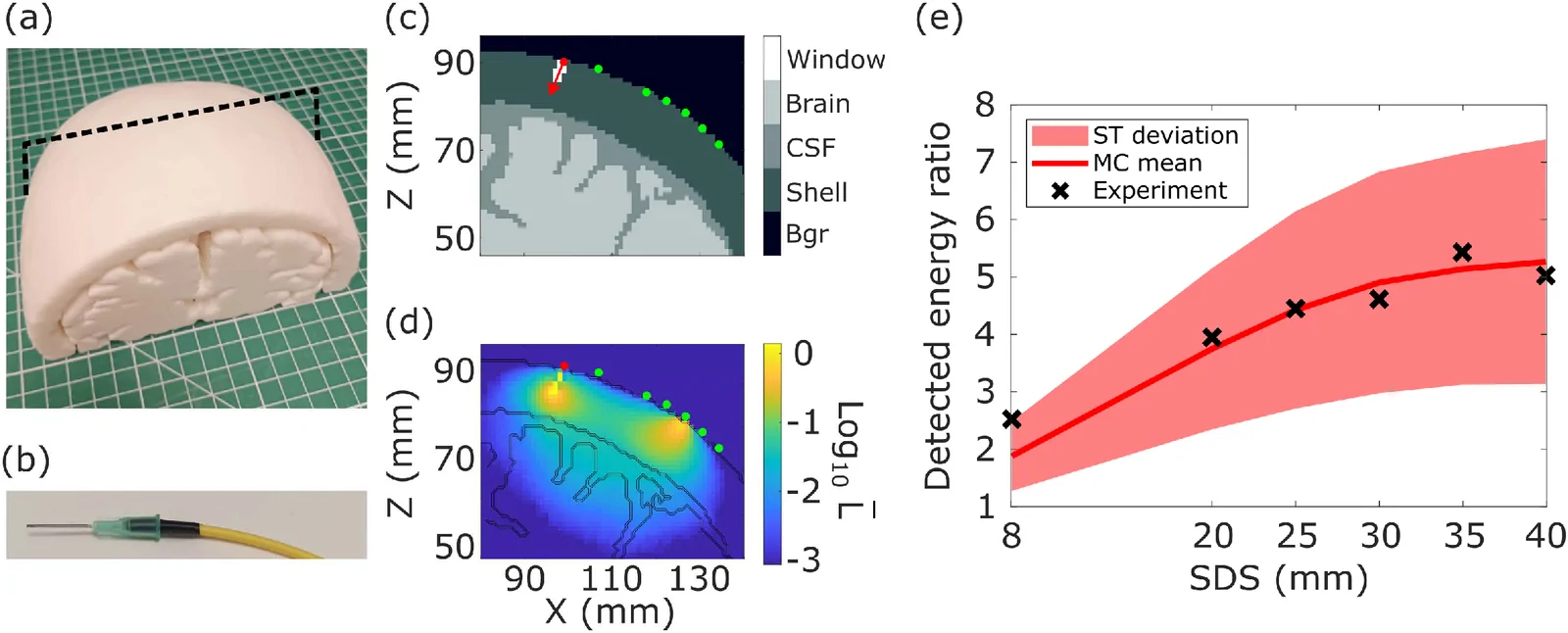
A 3D printed resin phantom (a) consisting of three layers: white resin brain and shell (combined scalp with skull) and transparent gelatine in between, and the modified optical fibre inserted into a hypodermic needle (b). The dashed line in (a) indicates an approximate location for volumetric (c) and sensitivity profile (d) cross sections. The red and green circles in (c) and (d) mark location of sources and detectors respectively. The red arrow in (c) shows source direction. In (e), a quantitative comparison of detected energy ratio between experimental measurements and numerical simulations. The uncertainty band indicates numerical detected energy ratio standard deviation of 7 X- and Y-translated (≤5mm) source-detector montage positions.

Figure 4(e) shows the comparison of the detected energy ratio as a function of SDS. The numerical mean values (MC mean) show a clear increasing trend with increasing SDS. The shaded red region represents 1 standard deviation from the mean obtained from multiple simulations as described above, and captures the variability in signal that one might expect as a result of the anatomical variability due to optode placement. The experimental measurements (black crosses) follow the simulated mean curve and lie within the simulated standard deviation range across all separations.

We note that whilst the absolute values of the white resin phantom simulated and experimental energy ratios agree, they are significantly smaller compared to the case of the anatomically more precise models used above. We ascribe this difference to the lower transmission of light when the full scalp/skull details are included in the model, which then implies a much higher gain factor when these are ‘bypassed’ with the use of the implants. Moreover, whilst the reduced scattering coefficient of the white resin is approximately similar to biological tissue, the absorption coefficient is 20-40× lower, which also leads to smaller energy ratios compared to the Colin27 simulations. As mentioned, the goal with this experimental study was to validate the numerics in a setting that could be precisely replicated in silico and in the lab and to this end, we opted for a more feasible, simplified 3-layer resin phantom model with accurate brain anatomy, instead of a phantom with fully realistic anatomical and tissue heterogeneity. We believe that the observed agreement in this setting lends some support to the notion that results in the previous sections might also then apply to the related real-world cases, ahead of performing actual human tests in the future.

## 4. Discussion and conclusions

Optical implants delay the onset of scattering effects for the input source light until it reaches the deeper layers of the head and brain. As expected from established photon transport principles, bypassing the highly scattering scalp and skull increases the fraction of photons interrogating cerebral tissue. The present work moves beyond this somewhat intuitive and obvious understanding and instead provides a quantitative engineering evaluation of minimally invasive optical implants, characterising their impact on detected photon energy and sensitivity profiles and providing guidance for the optimisation of implant design and placement across realistic anatomical models

At short separations (SDS = 8 mm) with the use of optical implants, sensitivity metrics related to the gray matter sensitivity show values comparable or higher than the typical standard fNIRS separations (SDS = 25-40 mm) without optical implants. This implies that the optical implants enable probing of GM at SDS that would otherwise be dominated by scalp signal (Fig. 2(c-f)). As the light reaches deeper regions of the head with optical implants, the optical path to the detectors becomes longer and the detected light becomes more attenuated. This could be seen as a benefit as source power in standard fNIRS for short channels needs to be adjusted or attenuated such that the detected signal would not saturate the detectors [27,28].

For longer channels, the introduction of optical implants can enhance "coupling" of light into the low-scattering CSF pathway, thereby improving guiding efficiency (see Fig. 1 and Supplement 1). For SDS > 25 mm this results in 2-3 orders of magnitude and 2-4 orders of magnitude improvement in energy transport (expressed as $E_{\text{ratio}}$ in Fig. 2(b)) for transdermal and transcranial cases, respectively.

Also of importance for practical considerations (where and how many implants to use), the higher energy transport and sensitivity gains are obtained with an implant only for the source, with smaller additional gains when adding a second implant at the detector side. The present study quantitatively characterises the non-trivial asymmetry between source- and detector-side implants, providing design guidance for optimising implant placement across realistic anatomical models.

For transdermal implants, the gain remains substantial for the source-only case with $E_{\text{ratio}}$ up to two orders of magnitude higher (for SDS≥30mm ) than the reference case. As this configuration requires only one implant and does not require penetration of the skull, it represents the least invasive option among those considered.

Although the present study considers only continuous-wave (CW) measurements, the proposed concept is equally applicable to TD-fNIRS. The primary advantage of the optical implant – bypassing the highly scattering scalp and skull – is independent of the detection modality and therefore benefits both CW and TD systems.

Generally, invasive neurotechnological measurements may not be acceptable or appealing to all potential users, motivating interest in less invasive or minimally invasive approaches. Established clinical procedures such as burr-hole craniotomy are already routinely used in intracranial EEG (iEEG), primarily for epilepsy monitoring, and represent a well-characterized example of implanted neural recording interfaces [29]. In such procedures, closure of the surgical site is typically straightforward, it is enough to use one stitch to close the incision. However, even in these controlled clinical settings, implantation involves surgical overhead and associated risks.

At the same time, the translational potential of such minimally invasive approaches should be considered cautiously. While transdermal piercings are widely used in non-medical contexts, their direct adoption as medical interfaces remains speculative. Any translation to clinical use would require careful evaluation of key challenges, including infection risk, long-term tissue response, fibrotic encapsulation, mechanical stability, and potential degradation of optical performance over time [30,31]. In addition, regulatory requirements and patient acceptance will play a critical role in determining feasibility.

In addition, a fixed implant could enable more repeatable optode placement, which is expected to be important for longitudinal monitoring and reproducibility. For example, mechanical anchoring might be expected to reduce motion artefacts, although at this stage this remains untested.

We finally note that similar results could also be obtained without physical implants. There are other options that allow one to temporarily (and perhaps in future, permanently) render the skin or bone transparent. Tissue transparency can for example be achieved by absorbing specific dye molecules or alcohols that modify the tissue properties, rendering it transparent to the injected light [15–17]. One may envisage a situation where such dyes or other materials are locally applied or injected into the scalp, thus achieving transparency. Currently, this is achieved for durations of 10-20 minutes before the dye needs to be washed away. Future development may lead to materials that will not need to be washed away and could achieve permanent transparency of the scalp and or bone.

## Supplemental information

Supplement 1 Supplementary material https://doi.org/10.6084/m9.figshare.33161747

## Data Availability

Data underlying the results presented in this paper are available at [32].

## Supplemental document

See Supplement 1 for supporting content.
