# Supplementary material for: Optical performance of minimally invasive brain implants: a simulation and phantom study
Source: Biomed Opt Express. 2026 Aug 12;17(9):4593–604. doi: 10.1364/BOE.604502 (PMC13592266; doi:10.1364/BOE.604502)
Supplement: Supplementary file 1 [file boe-17-9-4593-s001.pdf]

Supplemental Document

Biomedical Optics EXPRESS

Optical performance of minimally invasive brain implants: a simulation and phantom study: supplement

VYTAUTAS GRADAUSKAS, JEMMA NEIL, KEVIN J. MITCHELL, JACK RADFORD, AND DANIELE FACCIO*

School of Physics and Astronomy, University of Glasgow, Glasgow, UK
*daniele.faccio@glasgow.ac.uk

This supplement published with Optica Publishing Group on 12 August 2026 by The Authors under the terms of the Creative Commons Attribution 4.0 License in the format provided by the authors and unedited. Further distribution of this work must maintain attribution to the author(s) and the published article's title, journal citation, and DOI.

Supplement DOI: https://doi.org/10.6084/m9.figshare.33161747

Parent Article DOI: https://doi.org/10.1364/BOE.604502

Optical Performance of Minimally Invasive Brain Implants: A Simulation and Phantom Study Supplementary material

Vytautas Gradauskas, Jemma Neil, Kevin J. Mitchell, Jack Radford, and Daniele Faccio[*]

School of Physics and Astronomy, University of Glasgow, Glasgow, UK
[*]*daniele.faccio@glasgow.ac.uk*

Abstract: Supplementary information containing additional simulation results.

© 2026 Optica Publishing Group

1. Optical properties

We have compiled skin (scalp), skull, CSF, GM and WM absorption coefficient μ_a and reduced scattering coefficient $\mu_s' = \mu_s(1-g)$ spectra for visible (VIS) and near-infrared (NIR) wavelengths from commonly used references [1–7].

1.1. Absorption coefficient

Each tissue type absorption coefficient was a combination of molar extinction coefficients (HbO and HbR), water volume fraction, lipid volume fraction and additionally melanosome volume fraction for skin (scalp). The HbO and HbR molar extinction coefficient spectra, water and lipid absorption spectra were taken from Roblyer et al. [1]. Melanosome absorption spectra was modelled using Eq. 8 defined by Jacques [2].

Molar concentrations for HbO and HbR were taken from Doulgerakis et al. [3]. Note that for the CSF we did not use their proposed value and only used water absorption spectrum as CSF is 99 % water [4]. Water and lipid volume fractions were taken from Duck [4] and melanosome volume fraction from Roblyer et al. [1].

The compiled absorption spectra for skin (scalp), skull, CSF, GM and WM are shown in Fig. 1 (a). The vertical dashed line indicates μ_a values used in the simulations throughout the main manuscript for $\lambda = 850$ nm.

1.2. Reduced scattering coefficient

The reduced scattering coefficients for skin, skull and brain were modelled using Eq. 2 and Table 2 as reported by Jacques [2]. The reference reports a common μ_s' for brain tissue. We assigned this value to GM and set WM to three times higher, which is reasonable given experimental measurements indicating WM μ_s' are approximately 3.4–6 times greater than those of GM [5, 6].

The CSF was modelled with a constant $\mu_s' = 0.01$ mm^{-1} for the wavelength range as suggested by Strangman et al. [7].

The modelled reduced scattering spectra for skin (scalp), skull, CSF, GM and WM are shown in Fig. 1 (b). The vertical dashed line indicates μ_a values used in the simulations throughout the main manuscript for $\lambda = 850$ nm.

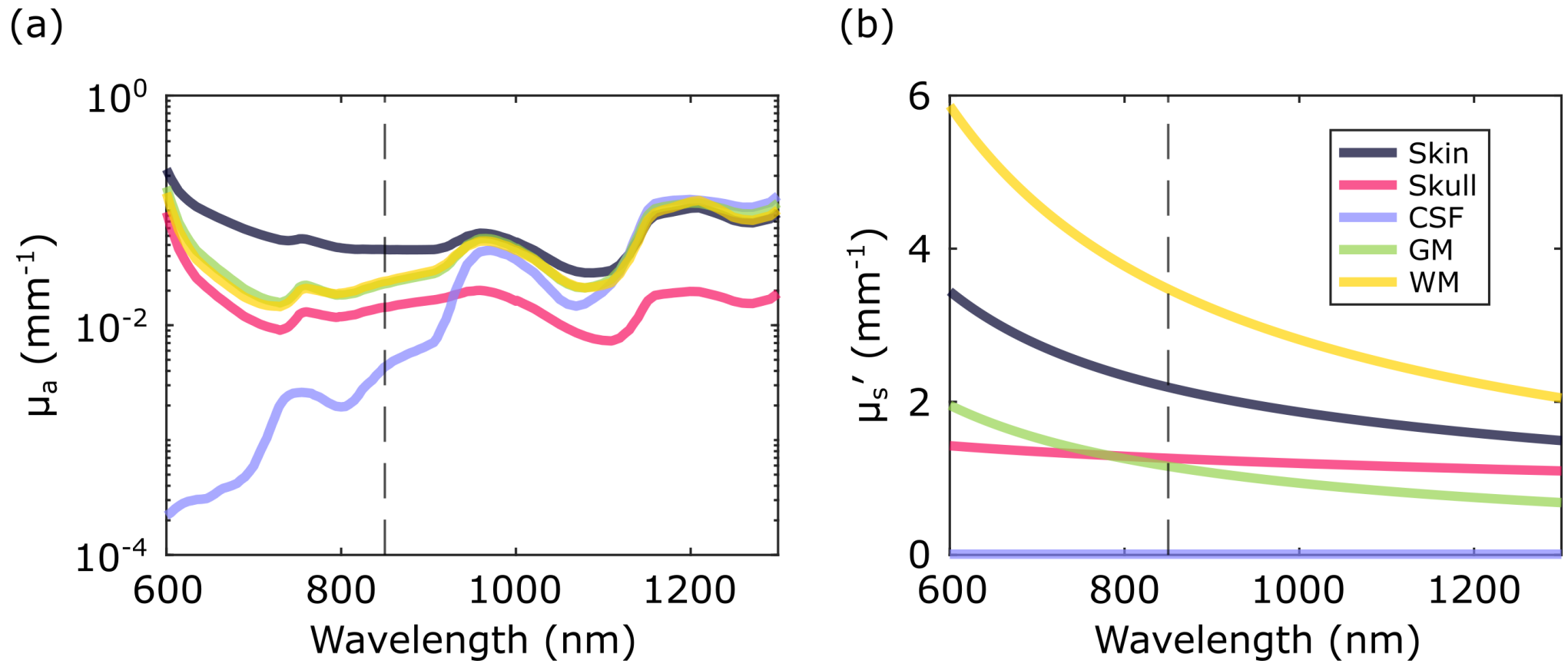

Fig. 1. Absorption (a) and reduced scattering (b) coefficient spectra for skin (scalp), skull, CSF, GM and WM. The dashed vertical line indicates λ = 850 nm.

2. Sensitivity profiles at SDS = 8 mm

The normalised logarithmic spatial sensitivity profiles for SDS = 8 mm are depicted in Fig. 2(c-g). Spatial sensitivity profiles for the remaining SDS are provided in the supplementary material. In the standard reference case, Fig. 2(c), the characteristic "banana" shape sensitivity profile penetrates only into the superficial scalp layer – this is typical for short-channels that are therefore typically used to monitor (and remove in post-processing) physiological signals [8, 9]. However, we see that higher sensitivity in deeper head regions can be achieved with transparent optical implant, Fig. 2(d-g), when compared to the standard reference case. Introducing a non-scattering optical implant for the source allows the light to reach deeper layers of the head before it starts to scatter in tissue. This strongly modifies the typical sensitivity profile: intuitively, a deeper implant increases sensitivity for deeper head layers, e.g., for GM, by up to 2 orders of magnitude. When both the source and detector use transcranial implants in Fig. 2(g), the highest sensitivity region follows a U-shape and suggests a potential for higher spatial localisation together with the higher detection sensitivity.

The percent values in Fig. 2(c-g) indicate fractional gray matter sensitivity F_{GM} as defined by Eq. (3). For the reference case the fractional gray matter sensitivity is negligible with only F_{GM} = 0.0003% while already the least invasive STD optical implant shows a 3-orders of magnitude increase with F_{GM} = 0.25%. The BTC case has the highest fractional GM sensitivity of F_{GM} = 10.9%.

3. Sensitivity profiles

Sensitivity profiles for all configurations and all SDS are presented in Fig. 3.

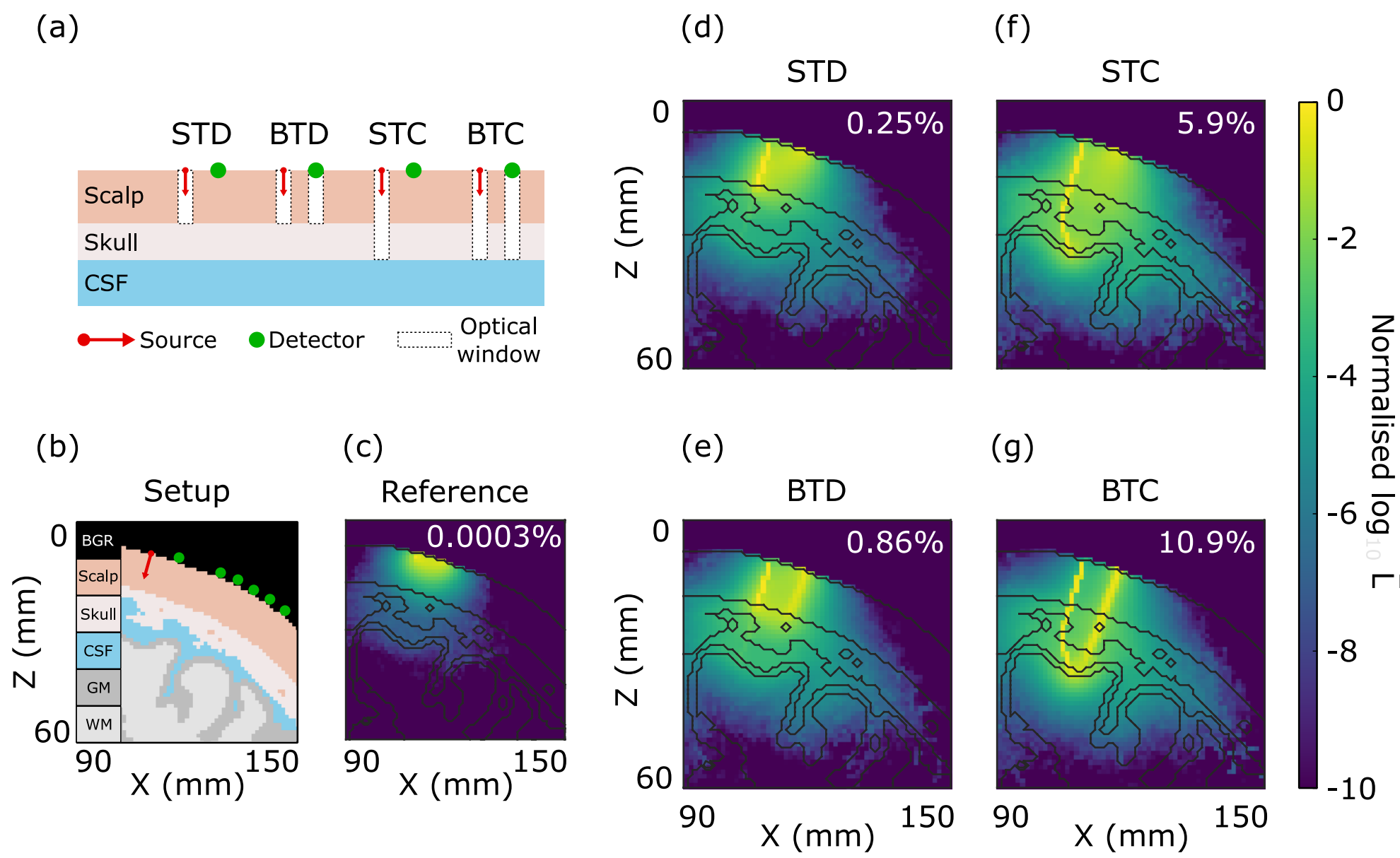

Fig. 2. A 2D graphical sketch of different transdermal and transcranial configurations (a). The abbreviations describe optical implants (white regions with dotted outline) for when the source alone is transdermal (STD), both source and detector are transdermal (BTD), the source is transcranial (STC) and both source and detector are transcranial (BTC) cases. Red arrows indicate the source position and direction. The green circles indicate detector positions. (b) The simulation setup showing the Colin27 head atlas cross section with different tissue types. The green circles mark the positions of 6 spherical detectors placed at SDS = 8, 20, 25, 30, 35, 40 mm. (c) Normalised logarithmic spatial sensitivity profiles for SDS = 8 mm for standard reference case and for modified regimes with: (d) transdermal source implant; (e) transdermal both source and detector implants; (f) transcranial source implant; (g) transcranial both source and detector implants. The percent values in (c-g) indicate fractional GM sensitivity (F_{GM}) as defined by Eq. (3). The colorbar is common for (c-g) spatial sensitivity maps and shows 10 orders of magnitude from the maximum value.

4. Enhanced scattering-guided light

Diffuse light can be guided through low-scattering channels (core) that are surrounded by high-scattering regions (cladding) [10]. Within the human head, the cerebrospinal fluid (CSF) layer may be interpreted as such a weakly scattering core, enclosed by highly scattering tissues, including the brain (grey matter and white matter) and extracerebral layers (skull and scalp). This structural configuration enables light to propagate over relatively long distances, e.g., diametrically across an adult human head [11], prior to detection. As this mechanism likely represents the least attenuating pathway for photon propagation in the head, maximizing the proportion of source light that reaches the CSF layer is desirable to facilitate preferential guidance along this channel.

The introduction of optical implants can enhance "coupling" of light into the low-scattering CSF pathway, thereby improving guiding efficiency (Fig. 4). This effect is also reflected in the spatial sensitivity profiles, as sensitivity is inherently determined by photons that successfully reach the detector. Preferential propagation along the CSF is particularly evident in the BTC configuration, where light predominantly follows the curved CSF geometry.

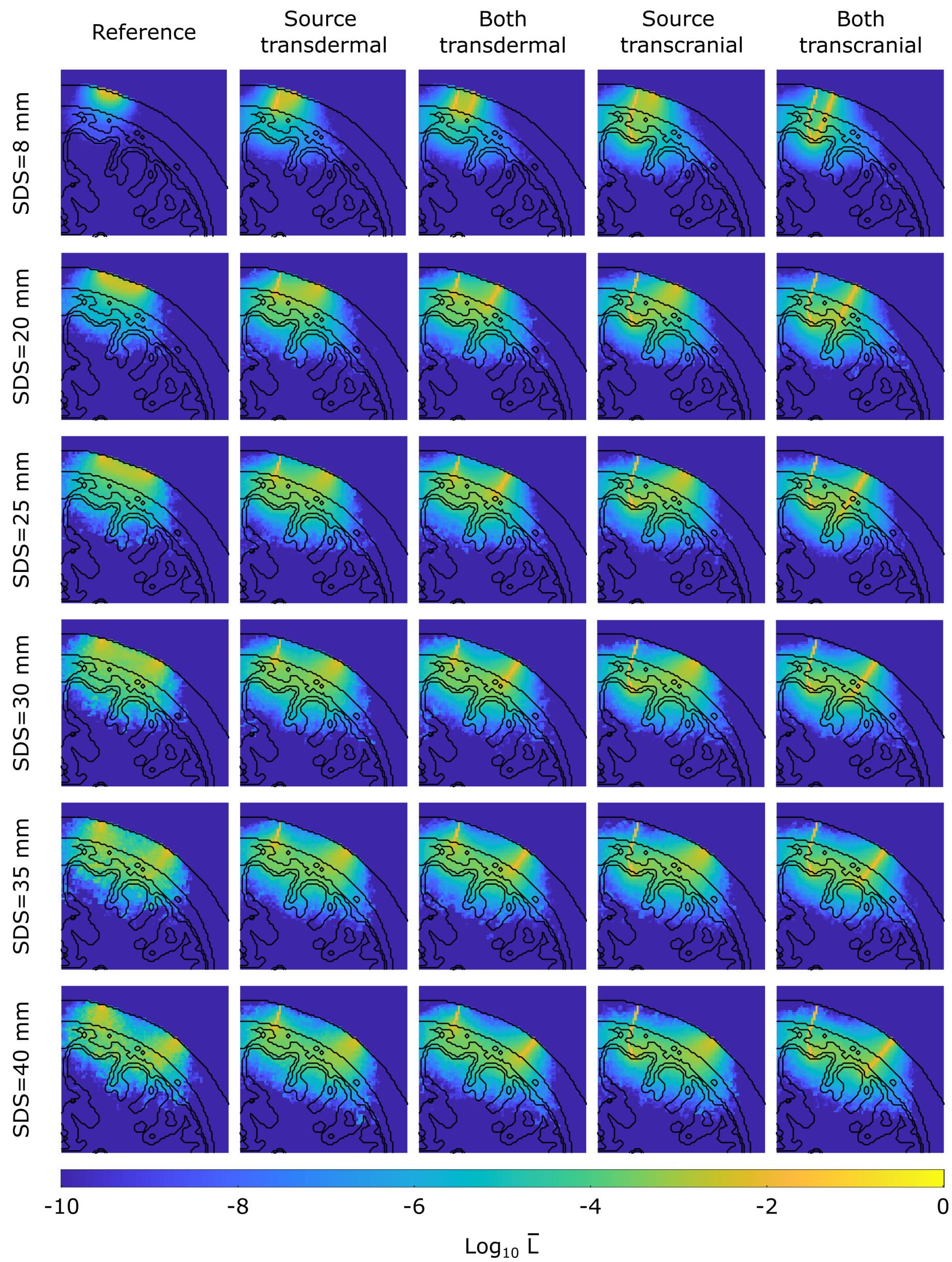

Fig. 3. Spatial $\log_{10}$ sensitivity profiles for reference (standard), source transdermal, both transdermal, source transcranial, both transcranial cases for all modelled SDS = 8, 20, 25, 30, 35, 40 mm. The colorbar shows 10 order of magnitude from the common maximum value.

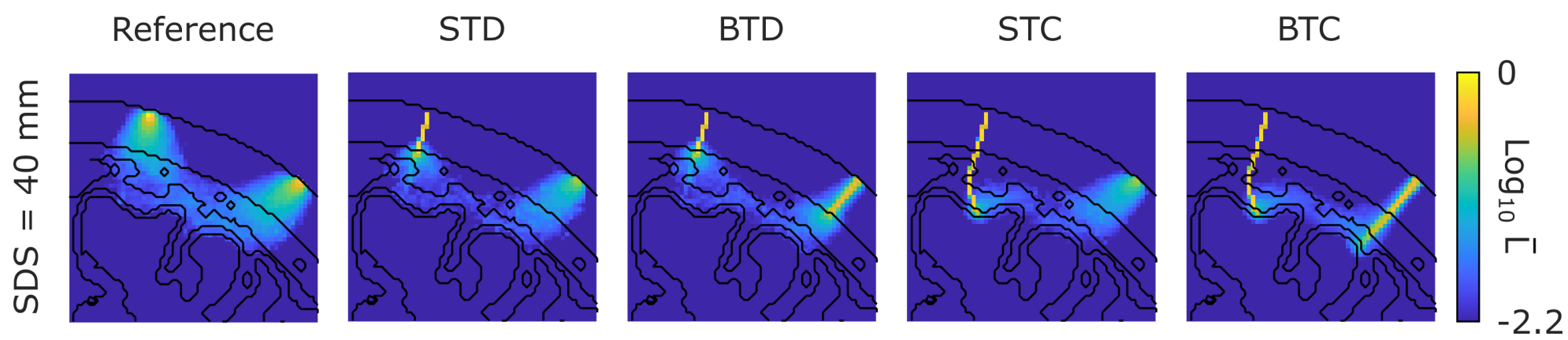

Fig. 4. Normalised logarithmic spatial sensitivity profiles for SDS = 40 mm for standard reference, source transdermal (STD), both transdermal (BTD), source transcranial (STC), both transdermal (BTC) configurations. The data was thresholded to emphasise the regions with the highest sensitivity.

5. Metrics

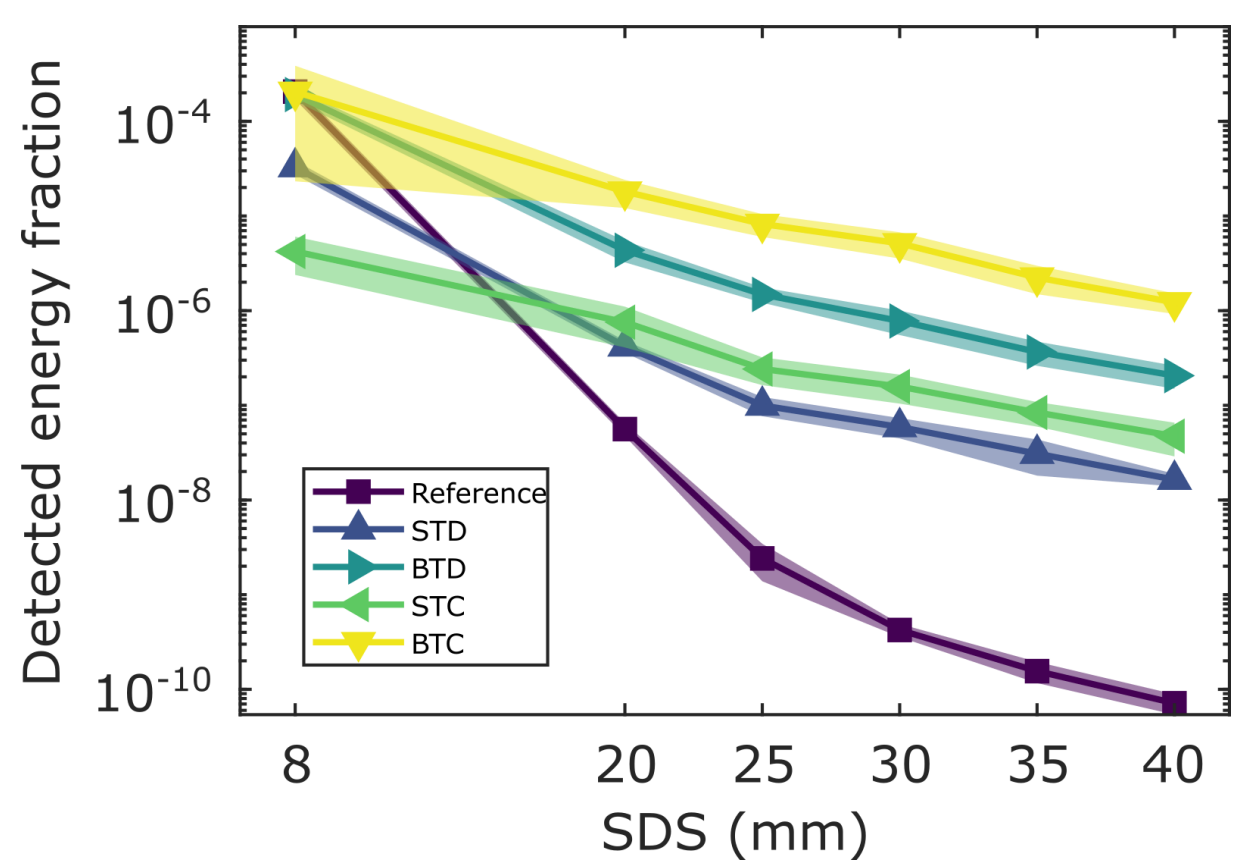

Fig. 5. Detected energy fraction for standard reference, source transdermal (STD), both transdermal (BTD), source transcranial (STC), both transdermal (BTC) configurations.

The absolute detected energy fractions for all cases are provided in Fig. 5.

6. Scalp dip

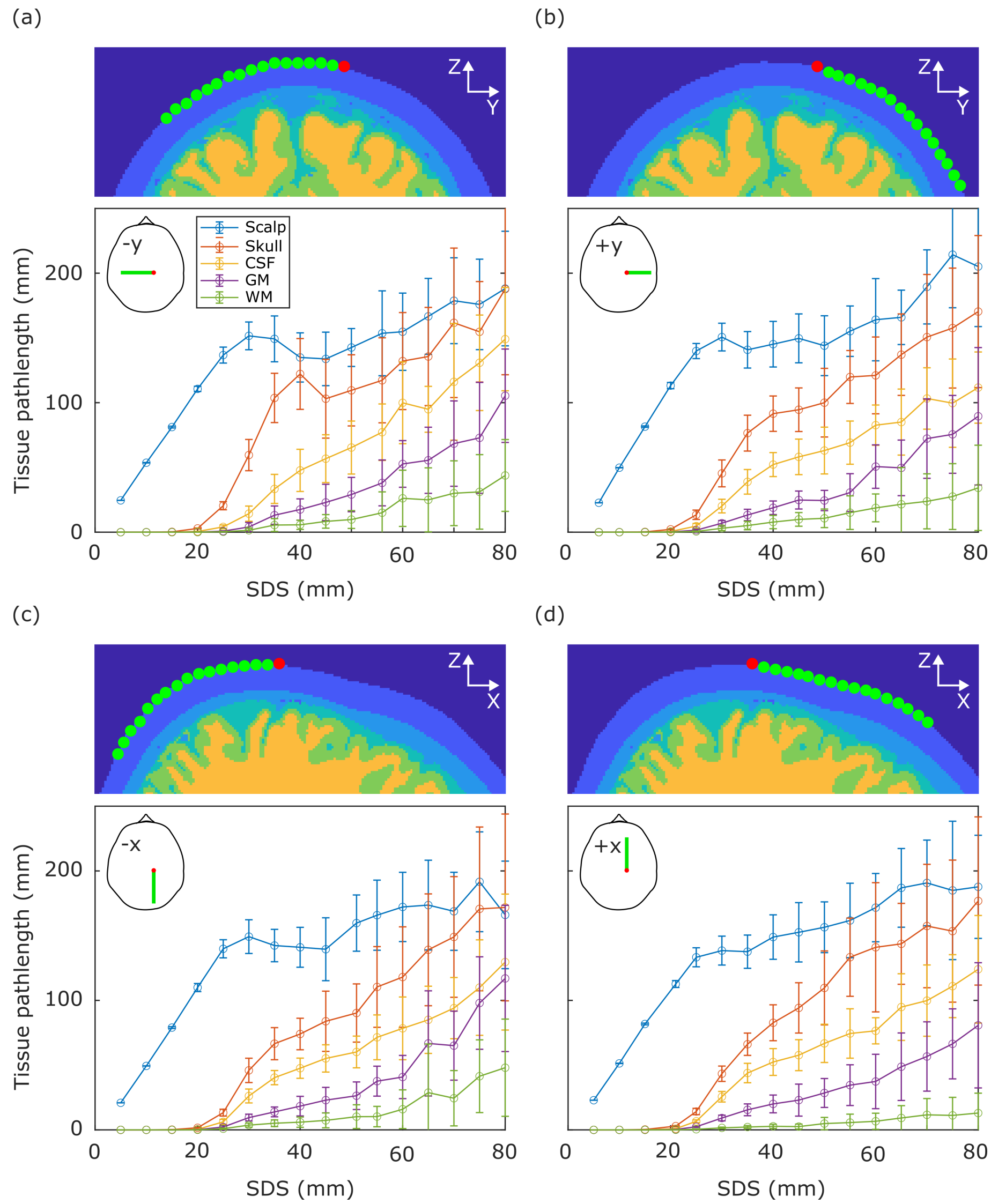

Fig. 6. (a-d) top anatomical structure images of Colin27 with source (red circle) and detector (green circles) placements for various montage positions. (a-d) tissue pathlength plots for various montage positions. The insets indicate approximate location of the linear montage as a top view of Colin27. The error bars are 1 standard deviation from the mean for 20 distinct photon packet launch seeds (20×10^8).

The simulation setup is the same as for the standard reference case in the main article, except the linear montage was set to have SDS linearly spaced between 5 mm and 80 mm in increments of 5 mm. For each simulation configuration the linear montage was rotated 90° around the source as shown in Fig. 6(a-d) top images and plot insets. A total number of 2×10^9 number of photon packets were launched for each of the simulation setup (divided by 20 different seeds). The plots in Fig. 6(a-d) show tissue pathlength for all SDS.

We observe a small dip in the scalp pathlength from around SDS = 30 mm. When changing

the orientation for the source-detectors along the scalp, the shape of this curve, including the presence of the dip itself is modified therefore hinting that this dip is linked to the specific skull (and overlaying scalp) shape and geometry along the source-detector direction.

7. Localisation of gray matter sensitivity for short-channel implants

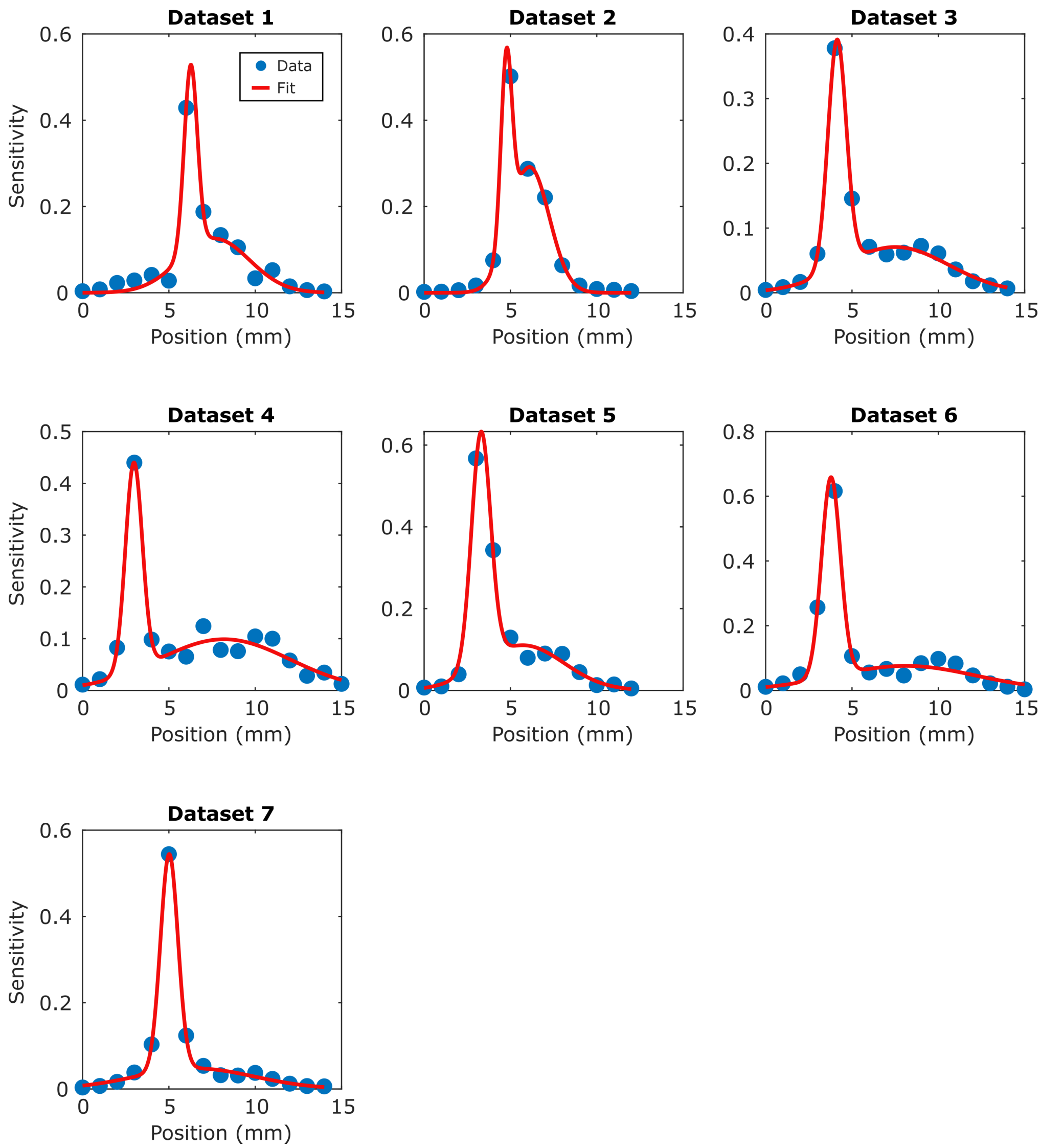

Fig. 7. The data (blue circles) are sensitivity values extracted along GM surface for BTC optical implant case for each linear montage position (7 datasets). The data was fitted with a double Gaussian curve (red line).

8. White resin optical coefficients

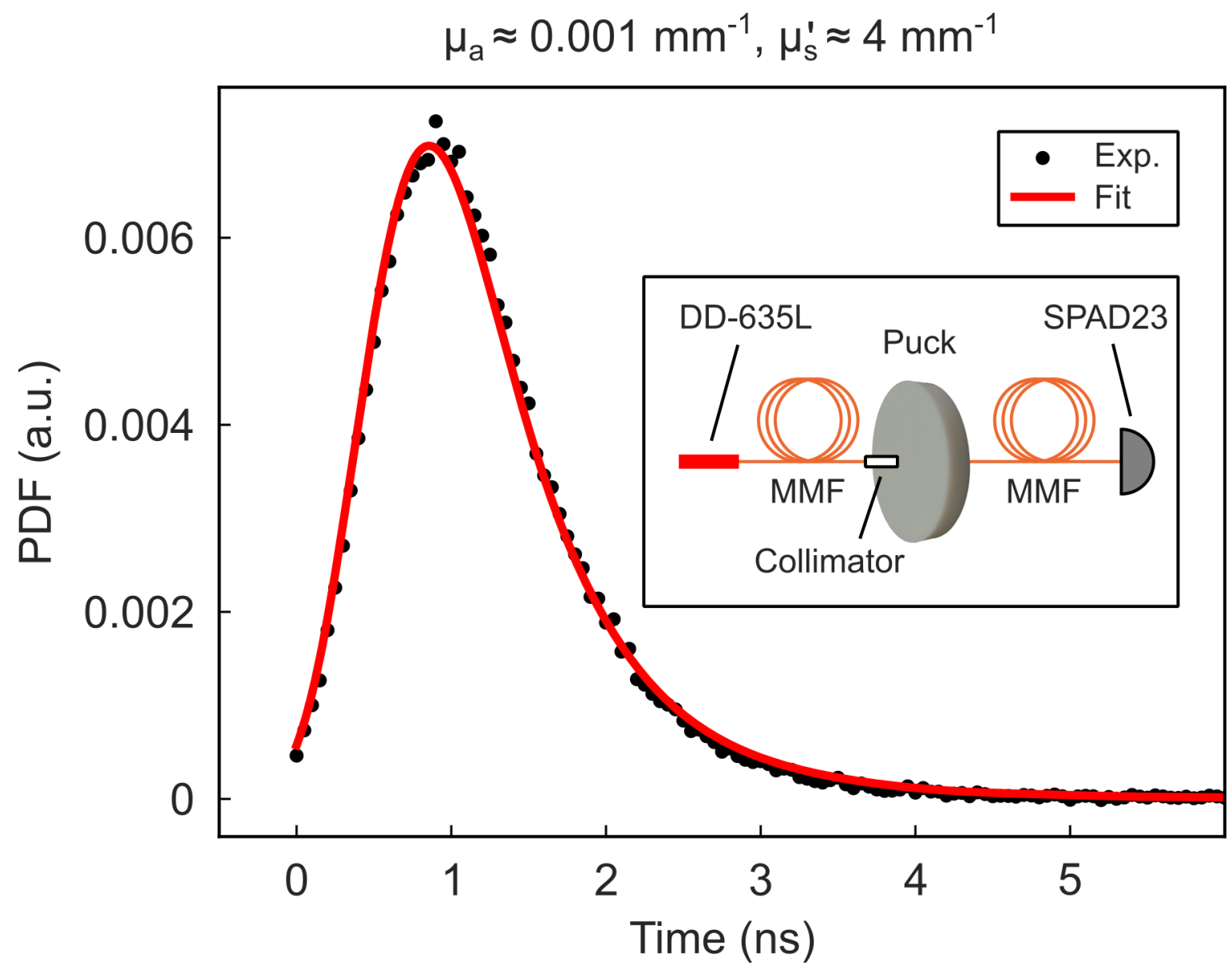

Fig. 8. Normalised experimental data (black dots) and forward model fit (red line) for white resin puck. The fitted values were $\mu_a \approx 0.001$ mm^{-1} and $\mu_s' \approx 4$ mm^{-1} for absorption and reduced scattering coefficients respectively. The inset shows experimental setup with

To characterise the 3D printed Formlabs V4.1 white resin, a reference sample in the form of a cylindrical puck was fabricated. The puck had a thickness of $z_{puck} = 12.5 \pm 0.02$ mm and a radius of $R_{puck} = 40 \pm 0.5$ mm. A schematic of the experimental arrangement is shown in Fig. 8 inset. Photon time-of-flight (ToF) measurements were carried out in transmission mode.

Excitation source was a pulsed HORIBA DeltaDiode laser (model DD-635L), operating at a central wavelength of 635 ± 10 nm with a pulse duration of 60–80 ps. The laser output was coupled into a multimode optical fibre equipped with a collimator. The resulting beam, with an approximate waist diameter of $d_{waist} \approx 2.1$ mm, was directed onto the centre of the sample. Transmitted photons were collected using a multimode fibre (core diameter 1 mm, numerical aperture 0.39), which was connected to a Pi Imaging SPAD23 detector array. Data acquisition was performed at a repetition rate of 40 MHz.

To determine the instrument response function (IRF), the sample was replaced with a sheet of paper and the laser intensity was reduced. This approach, involving a highly scattering medium such as paper or Teflon tape, is commonly used because it does not introduce significant temporal broadening of the pulse [12, 13]. The measured ToF data, after background subtraction and normalisation, are presented as black markers in Fig. 8. The data was cropped at early times corresponding to the IRF peak, and at late times where the signal approached zero.

A forward model of the experiment was implemented using MCX. In the simulation, a uniform, collimated circular source with diameter $d_{src} = 2$ mm illuminated a cylindrical domain matching the physical sample dimensions ($z = 12.5$ mm, $R = 40$ mm). Photon packets in transmission were detected by a spherical detector of radius $d_{det} = 1$ mm, positioned on the surface opposite the source. The simulated photon ToF distribution was convolved with the experimentally measured IRF.

The refractive index was fixed at $n = 1.505$, based on reported values [14], and the scattering

anisotropy was set to $g = 0.9$, consistent with typical values for biological tissue in the red and near-infrared spectral range [2]. The absorption coefficient μ_a and scattering coefficient μ_s were estimated via a least-squares optimisation using the `optimize.curve_fit()` function from the SciPy library.

The fitted parameters were found to be approximately $\mu_a \approx 0.001$ mm^{-1} and $\mu_s \approx 40$ mm^{-1}. From these, the reduced scattering coefficient was calculated as $\mu_s' = \mu_s(1-g) = 40\times(1-0.9) = 4$ mm^{-1}. The resulting fit is shown as a red curve in Figure 8.

9. Comparison with Beer-Lambert law

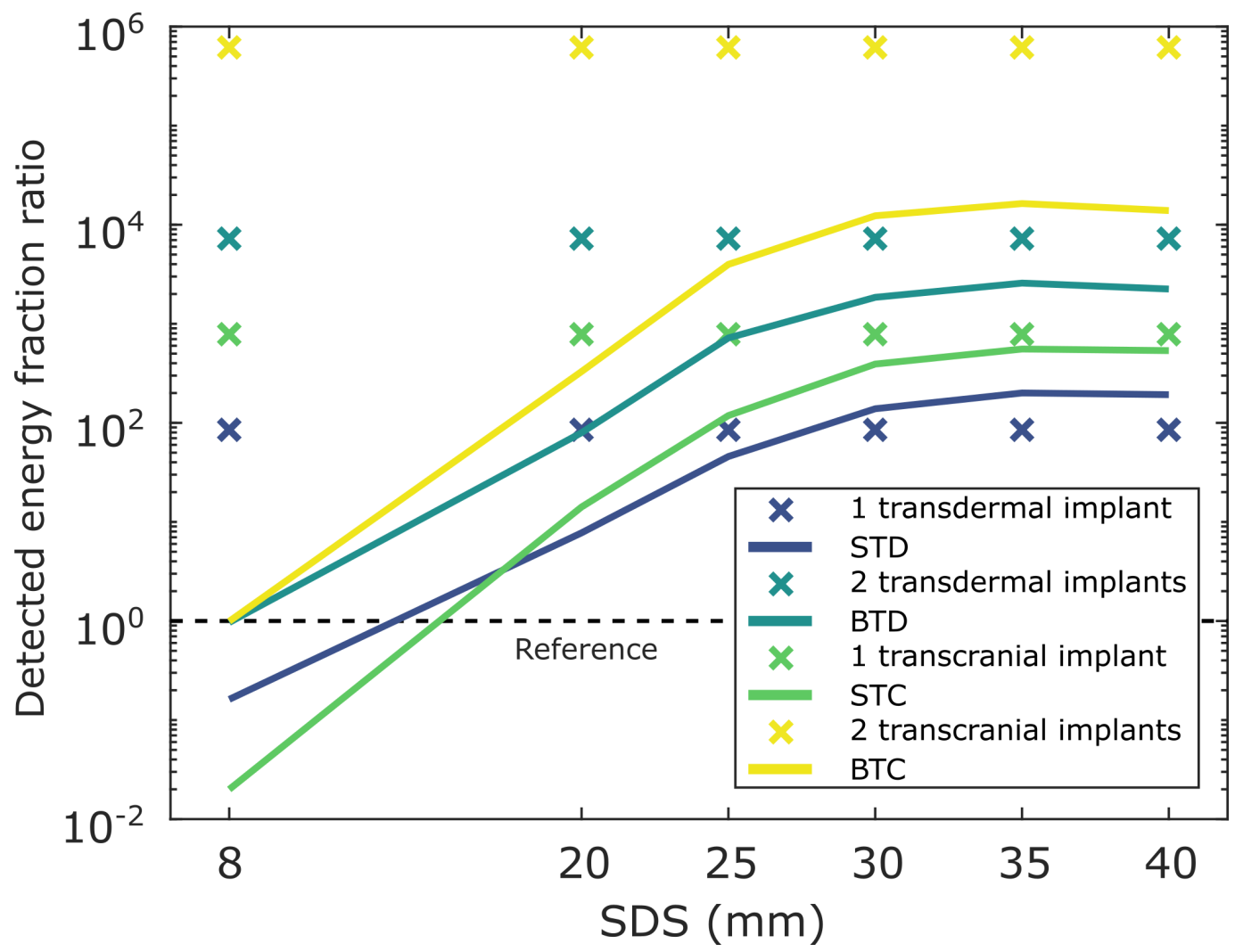

Fig. 9. Detected energy factor ratio comparing analytical calculations (crosses) using Beer-Lambert law (Eq. (1) and Eq. (2)), and numerical simulations (solid lines) described in the main manuscript.

Back-of-the-envelope calculations using Beer-Lambert law would not be sufficient to determine the optical implant effect. We calculate the expected attenuation factor for scalp parameters: $\mu_s' = 2.19$ mm^{-1}, $\mu_a = 0.046$ mm^{-1}, $\mu_{eff} = \sqrt{3\mu_a(\mu_a + \mu_s')} = 0.556$ mm^{-1}, $DPF = 6.5$ [15], SDS = 8, 20, 25, 30, 35, 40 mm. For the reference case:

$$Ref = \exp(-\mu_{eff}(DPF \times SDS)) \tag{1}$$

If we consider implants with lengths $z_{implant} = 8$ mm (transdermal) and $z_{implant} = 12$ mm (transcranial), then attenuation factor becomes:

$$Implant = \exp\Big(-\mu_{eff}\big((DPF \times SDS) - z_{implant}\big)\Big) \tag{2}$$

The analytical detected energy factor ratio comparing implant (Eq. (2)) and reference (Eq. (1)) cases is shown in Fig. 9, crosses. Using Beer-Lambert law only captures a uniform increase across SDS in detected energy ratio which is the most similar to long separations (e.g., SDS = 40 mm) in the numerical simulations (Fig. 9, lines), where the energy fraction ratios have plateaued. This shows that a simple diffuse model cannot capture the more complex behaviour of light propagation, especially at short channels (SDS = 8 mm).

References

1. D. Roblyer, A. Pilvar, T. Pham, *et al.*, "Review of shortwave infrared imaging and spectroscopy in tissue," Biomed. Opt. Express **16**, 5028–5062 (2025).
2. S. L. Jacques, "Optical properties of biological tissues: a review," Phys. Medicine & Biol. **58**, R37 (2013).
3. M. Doulgerakis, A. T. Eggebrecht, and H. Dehghani, "High-density functional diffuse optical tomography based on frequency-domain measurements improves image quality and spatial resolution," Neurophotonics **6**, 035007–035007 (2019).
4. F. Duck, *Physical Properties of Tissue: A Comprehensive Reference Book* (Academic Press, 1990).
5. J. Shapey, Y. Xie, E. Nabavi, *et al.*, "Optical properties of human brain and tumour tissue: an ex vivo study spanning the visible range to beyond the second near-infrared window," J. biophotonics **15**, e202100072 (2022).
6. A. Yaroslavsky, P. C. Schulze, I. V. Yaroslavsky, *et al.*, "Optical properties of selected native and coagulated human brain tissues in vitro in the visible and near infrared spectral range," Phys. Medicine & Biol. **47**, 2059 (2002).
7. G. Strangman, M. A. Franceschini, and D. A. Boas, "Factors affecting the accuracy of near-infrared spectroscopy concentration calculations for focal changes in oxygenation parameters," Neuroimage **18**, 865–879 (2003).
8. L. Gagnon, K. Perdue, D. N. Greve, *et al.*, "Improved recovery of the hemodynamic response in diffuse optical imaging using short optode separations and state-space modeling," Neuroimage **56**, 1362–1371 (2011).
9. S. Brigadoi and R. J. Cooper, "How short is short? optimum source-detector distance for short-separation channels in functional near-infrared spectroscopy," Neurophotonics **2**, 025005–025005 (2015).
10. K. J. Mitchell, V. Gradauskas, J. Radford, *et al.*, "Energy transport in diffusive waveguides," Nat. Phys. **20**, 1955–1959 (2024).
11. J. Radford, V. Gradauskas, K. J. Mitchell, *et al.*, "Photon transport through the entire adult human head," Neurophotonics **12**, 025014 (2025).
12. H. Wabnitz, D. R. Taubert, M. Mazurenka, *et al.*, "Performance assessment of time-domain optical brain imagers, part 1: basic instrumental performance protocol," J. biomedical optics **19**, 086010–086010 (2014).
13. J. Carlsson, P. Hellentin, L. Malmqvist, *et al.*, "Time-resolved studies of light propagation in paper," Appl. optics **34**, 1528–1535 (1995).
14. G. D. Berglund and T. S. Tkaczyk, "Fabrication of optical components using a consumer-grade lithographic printer," Opt. Express **27**, 30405–30420 (2019).
15. A. Duncan, J. H. Meek, M. Clemence, *et al.*, "Optical pathlength measurements on adult head, calf and forearm and the head of the newborn infant using phase resolved optical spectroscopy," Phys. Medicine & Biol. **40**, 295–304 (1995).
